# Strengthening translational preclinical research through confirmatory multi-laboratory studies

**DOI:** 10.3389/fmed.2025.1715361

**Published:** 2026-01-07

**Authors:** Sophia C. Rotter, María Arroyo-Araujo, Natascha I. Drude, Pasquale Pellegrini, Sebastian Kobold, Günther H. S. Richter, Oliver J. Müller, Dunja Bruder, Lars B. Riecken, Björn Gerlach, Lena Schuler, Juliane Salbach-Hirsch, Iman Dalloul, Sebastian Kühn, Juliane C. Wilcke, Ulf Toelch

**Affiliations:** 1 QUEST Center for Responsible Research, Berlin Institute of Health at Charité—Universitätsmedizin Berlin, Berlin, Germany; 2 Division of Clinical Pharmacology, Ludwig-Maximilians-Universität (LMU) University Hospital, LMU Munich, Munich, Germany; 3 German Cancer Consortium (DKTK), Ludwig-Maximilians-Universität (LMU) University Hospital, LMU Munich, Munich, Germany; 4 Einheit für Klinische Pharmakologie (EKLiP), Helmholtz Zentrum München—German Research Center for Environmental Health Neuherberg, Munich, Germany; 5 Department of Pediatrics, Division of Oncology and Hematology, Charité—Universitätsmedizin Berlin, Berlin, Germany; 6 Department of Internal Medicine V, University Hospital Schleswig-Holstein, University of Kiel, Kiel, Germany; 7 German Centre for Cardiovascular Research (DZHK), Partner Site Hamburg/Kiel/Lübeck, Kiel, Germany; 8 Faculty of Medicine, Infection Immunology Group, Institute of Medical Microbiology and Hospital Hygiene, Otto-von-Guericke University Magdeburg, Magdeburg, Germany; 9 Research Group Immune Regulation, Helmholtz Centre for Infection Research, Braunschweig, Germany; 10 Leibniz Institute on Aging, Fritz Lipmann Institute, Jena, Germany; 11 Institute of Psychopharmacology, Central Institute of Mental Health, Mannheim, Germany; 12 Guarantors of EQIPD e.V. Heidelberg, Heidelberg, Germany; 13 PAASP GmbH, Heidelberg, Germany; 14 Department of Paediatrics, Children’s Cancer Research Centre, Kinderklinik München Schwabing, TUM School of Medicine, Technical University of Munich, Munich, Germany; 15 Medical Faculty at University Hospital Carl Gustav Carus, Dresden University of Technology, Dresden, Germany; 16 Division Polymer Biomaterials Science, Leibniz Institute for Polymer Research Dresden, Dresden, Germany; 17 Institute for Medical Information Processing, Biometry, and Epidemiology, Faculty of Medicine, LMU Munich, Munich, Germany

**Keywords:** preclinical research, confirmatory studies, multi-laboratory trials, reproducibility, stakeholder engagement, experimental design

## Abstract

Successful translation of promising preclinical findings into clinical application remains challenging. To address the rising concerns of failing clinical trials and the resulting economic, social, and ethical consequences, preclinical confirmatory studies have been proposed to generate sufficiently robust evidence for guiding the decision-making process. In a unique funding call, 17 studies in Germany aimed to confirm exploratory findings across various biomedical research fields in a rigorously planned and executed multi-laboratory set-up. Alongside these preclinical research projects, a meta-research project was funded to provide methodological support and collectively investigate confirmatory study design and experimental outcomes. After the first four-year funding period, an in-person workshop brought together representatives from the preclinical confirmatory studies to discuss lessons learned. We summarize the outcomes of these stakeholder discussions, highlight common pitfalls, and propose optimization strategies for experimental set-up and project coordination. As a result, we advocate for new roles—such as preclinical research coordinators—and improved rules and regulations in preclinical research to facilitate large-scale academic research projects. Moreover, we highlight that diverse stakeholders must collaborate to effectively integrate confirmatory multi-laboratory studies into the preclinical research ecosystem.

## 1 Introduction

Preclinical research seeks to generate evidence on the safety, efficacy, and mechanisms of novel interventions. Despite advancements in technology and major breakthroughs in patient-relevant discoveries, translational failures are still common where promising therapies fail to demonstrate efficacy in humans (1–4). These setbacks do not only pose serious health risks to patients who may receive new treatments with little to no benefit or worse, unforeseen harmful side effects (1), but also strain financial and healthcare systems (2, 5, 6). Moreover, as preclinical research predominantly relies on animal experiments to evaluate new interventions, studies that are poorly designed, underpowered, or use models that fail to reflect physical and biochemical characteristics of human disease, waste resources and raise ethical concerns (7). The drug development process from preclinical testing to clinical trial success is thus lengthy, uncertain and often high-risk. Consequently, existing strategies must be refined and extended to guide the decision-making in preclinical research towards increased efficiency.

One such extension involves conducting preclinical confirmatory studies as a decisive evaluation step before advancing to clinical trials (8–10). These studies help strengthen the evidence basis and narrow down the number of potential drug candidates, for instance (11). How does a confirmatory study differ from replication and exploratory studies? Most preclinical studies aim to generate hypotheses, making them inherently exploratory (12, 13). Relying on flexible experimental setups and different methodologies, these single-laboratory studies often lack transparency and sufficient standardization in study design, power calculations, statistical analyses, and reporting—frequently resulting in biased, underpowered, and poorly replicable findings (14–16).

In contrast, replication and confirmation studies adhere to rigorous methods mirroring those of clinical trials, including randomization, blinding, and adequate sample size calculations (12, 17). Whereas replication studies aim to repeat a previous experiment or study using the *same* methods and procedures to assess the reliability of the original result (18), confirmatory studies seek to validate, modify, or refute an existing knowledge claim by *systematically altering* certain aspects of the original experiment, thereby increasing the external validity of findings (9, 10, 12).

Another way to strengthen the external validity of findings is to conduct experiments across multiple laboratories (multi-laboratory study). Several preclinical multi-lab studies have demonstrated how these rigorous efforts yield more reliable, externally, and internally valid findings (19–21). A systematic assessment of preclinical multi-lab studies showed that these studies often produce smaller, more realistic effects sizes compared to exploratory single-lab studies (22). Furthermore, they often yield null or mixed results, demonstrating that multi-lab approaches better reflect true biological variance (22). Despite these successes, some multi-lab studies have also faced major challenges, for instance, in harmonizing and adhering to protocols across labs (21).

To encourage preclinical confirmatory multi-lab studies and explore their value, the German Federal Ministry of Research, Technology and Space (BMFTR) announced a unique funding line (23, 24). Seventeen funded projects performed rigorous efficacy studies to confirm previous exploratory findings across diverse biomedical research fields (psychiatry, oncology, immunology, cardiology, neurology, orthopedics, gastroenterology, urology). Each project conducted a number of experiments including a designated *in vivo* experiment across at least two partner laboratories at national or international level. Aiming at translation, these consortia implemented practices that enhanced both the validity (internal, external, translational) (9), and reliability of their results. Concretely, project groups followed harmonized protocols across participating labs, applied strategies to reduce risk of bias (e.g., randomization and blinding), and calculated required sample sizes *a priori* (25). Furthermore, some groups refined their disease models by, for instance, including both animal sexes or adopting more standardized induction techniques (25, 26). After the first four-year funding period, consortium representatives were invited to participate in an in-person workshop hosted by the *DECIDE* (Decision-Enabling Confirmation of Innovative Discoveries and exploratory Evidence) project team at the BIH QUEST Center for Responsible Research of the Berlin Institute of Health (BIH) at Charité—Universitätsmedizin Berlin. The *DECIDE* project, funded under the same BMFTR calls, provides methodological support for the confirmatory studies throughout the funding period while also analyzing study design, conduct, and outcome from a meta-research perspective (27, 28). By convening preclinical researchers, biostatisticians, quality management experts, clinicians, veterinarians, and representatives from the project management agency, the group jointly identified and discussed challenges and success factors of conducting preclinical confirmatory studies. In this article, we summarize the results of these stakeholder discussions and propose actionable recommendations. Whereas no single strategy fits all scenarios, this overview intends to inform the current state of knowledge based on the community’s experience to drive future advancements in preclinical research.

## 2 Confirmatory studies—common pitfalls and solution strategies

Although consortia varied in disease area, primary outcome, study design, methods, and models used, they all encountered similar challenges during the first funding period. Table 1 provides an overview of common pitfalls in the experimental set-up and project coordination of preclinical confirmatory multi-laboratory studies, along with proposed solution strategies derived from stakeholder discussions. The following sections explore overarching themes in greater detail. To illustrate some of the potential challenges involved in planning and conducting a preclinical confirmatory study, we present a fictionalized case study in Box 1. This example synthesizes experiences drawn from the actual confirmatory studies conducted during the first funding period without disclosing identifiable project details.

**Table 1:** Overview of common pitfalls and proposed optimization strategies for the experimental set-up and project coordination in preclinical confirmatory multi-lab studies, with selected practical resources and tools.

Aspect	Common pitfall	Optimization strategy	Resources and tools
Experimental set-up			
1. Centralizing procedures			
1.1 Protocol harmonization	Unexpected failure of established protocols at participating labs. Challenging implementation of procedures across labs, particularly for highly experimental or specialized methods. Variations in housing, handling, and breeding may not always be adaptable.	Central drafting and distribution of comprehensive and detailed protocol documents (e.g., SOPs, decision trees, videos). Decentralized discussion and feedback if necessary. Standardized training or workshops for staff of participating labs. Pilot study / test runs to validate protocols. Centralized support team / coordinating center for monitoring and oversight.	EQIPD Toolbox (54) PREMIER quality system (31, 52)
1.2 Compound preparation	Improper storage conditions. Inadequate transport and handling (e.g., compromising shipping conditions, delays, exposure to environmental factors). Discontinuation of original compound.	Standardized protocols and decision trees for storage, handling, and quality controls. Training of staff. Contingency plans for delays and other shipping disruptions. Reporting system to document changes (e.g., amend pre-registration).	ARRRIVE 2.0 (42) See also [5. Pre-Registration]
1.3 *In vitro/in vivo* experiments	Differences in equipment, technologies, standardization, and training across labs. Varying local regulatory requirements. Inconsistent reproducibility (e.g., due to variability in technique, execution, environmental factors including temperature, humidity, light).	SOPs and decision trees for standardization of key instruments, reagents, animal experimentation techniques. Central distribution of animals, materials, etc.; alternatively, parallel procurement / acquisition (e.g., ordering the same materials from same sources). Training of staff. Pilot studies / test runs. Regular monitoring and quality controls. Introducing systematic variation in housing conditions to counter the idiosyncratic conditions at each lab.	SOP templates for animal experimentation (55)
1.4 Blinding	Blinding not feasible. Inconsistent / improper blinding in one lab or across labs. Unintentional or necessary unblinding events (e.g., in case of information leaks, adverse events and/or serious complications). Non-transparent monitoring.	Automated blinding and randomization processes. Separating study arms to minimize accidental unblinding. Delegating critical tasks (e.g., model induction, confirmatory analyses) to independent personnel. SOPs, guidelines or decision trees for (un)blinding procedures. Centralized tracking system to monitor blinding across labs (e.g., digital tools, databases using barcodes or coded identifiers). Storage of controls / samples / specimens for potential verification of blinding at a later stage.	EQIPD Toolbox (54) EQIPD Template for Blinding Procedures (56) Experimental Design Assistant (EDA) (43) REDCap (41)
1.5 Randomization	Inconsistent randomization across labs (due to, e.g., technological, practical, or regulatory constraints). Complex urgent unblinding procedures.	Centralized randomization protocol and decision trees for unblinding. Training of staff. Centralized documentation,real-time monitoring, and interim analyses.	RandoMice (57, 58) Research Randomizer (59) QuickCalcs (60) Experimental Design Assistant (EDA) (43)
1.6 Quality management	Inconsistent compliance across labs throughout the project (e.g., due to limited or inefficient training, oversight, monitoring, communication, resources, or change of plans). Lack of sufficient quality controls across labs (e.g., manipulation checks).	Adhering to Good Laboratory Practice (GLP), additional support of GLP facilities. Centralized Quality Management Plan (QMP) with clearly defined metrics• Standardized data entry templates. Training of staff. Centralized oversight and coordination (e.g., via project management team, coordinating center). Reporting guidelines Predefined quality assurance measures	Principles of Good Laboratory Practice and Compliance Monitoring (36) PREMIER quality system (31, 52) EQIPD Toolbox (54) ARRRIVE 2.0 (42) RIVER recommendations (61) EQUATOR Network Reporting Guidelines (62)
2. Statistics			
	Inadequate sample size calculation and statistical models. Inadequate or non-transparent handling of missing data and dropouts. Unexpected data variability, adverse effects, “outlier” labs.	Early involvement of (and regular support from) (bio)statistician for power analysis and study design. Consultation with other stakeholders (e.g., clinicians, scientists, patient representatives). Pre-defined Statistical Analysis Plan (SAP) and Missing Data Plan with clearly pre-defined inclusion and exclusion criteria (e.g., in pre-registration). Independent statistical reviewer (e.g., DECIDE). Reporting guidelines for transparency	GPower (63) Computation of Effect Sizes (64) Experimental Design Assistant (EDA) (43) ARRRIVE 2.0 (42) Statistical pre-registration (65)
3. Data Management			
3.1 Data collection	Inconsistent data entry and quality. Differences in data formatting, software, databases, and equipment across labs.	Centralized Data Management Plan (DMP) following FAIR principles. Centralized data integrity checks with predefined parameters and threshold limits. Centralized platform (e.g., web-based cloud server). Use of widely accepted/standardized formats (to streamline formatting or archive integration with project nomenclature for files	FAIR principles (40) REDCap (41) Data Stewardship Wizard (66)
3.2 Data sharing (across labs)	Regulatory constraints (e.g., each institution may have different policies on intellectual property [IP], data ownership, usage rights). Logistical and technical issues (e.g., limited interoperability, large datasets, complicated data transfer). Data loss.	Pre-defined Data Sharing Agreements on IP, data ownership, and usage rights. Cloud-based data sharing platforms, repositories. DMPs and Data Transfer Protocols.	
4. Study Complexity			
	Defining and adhering to only one primary outcome. Poor correlation with clinical outcomes. Balancing rigor with feasibility given limited resources (time, budget, personnel). Overambitious study goals.	Clear endpoint selection and prioritization a-priori (e.g., pre-registration). Consultation with other experts (e.g., scientists, clinicians, patients or patient representatives). Pilot studies / test runs. Pooling of resources, partial segmentation of experimental set-up (e.g., regarding secondary endpoint analyses). Conduct regular progress reviews (e.g., by study coordinator) and adapt flexibly to any changes. Openness to adapt state-of-art procedures and medication.	PREPARE guidelines (39) Experimental Design Assistant (EDA) (43)
5. Pre-Registration			
	Time and resource constraints. Inadequate or unclear description of procedures. Low motivation (e.g., lack of incentives, fear of being scooped, unattractiveness of fixing seemingly irrelevant details). Limited availability of templates or guidance.	Use of existing templates to streamline process Training and support (online resources, workshops). Embargoed pre-registrations. Allocated time for pre-registration amendments	PreclinicalTrials.eu (67) AsPredicted.org (68) Animalstudyregistry.org (69) Open Science Framework (29, 70, 71)
Project coordination			
(A) Collaboration and Communication			
	Conflicting priorities. Uneven contributions or efforts across labs. IP, ownership, publication, and authorship issues. Unequal funding and resource distribution. Lack of clear communication.	Consortium agreement prior to study begin with clearly defined roles and responsibilities. Pre-defined agreements and guidelines regarding IP, ownership, publication, authorship with regular review and updates. Collaborative planning and allocation of resources. Comprehensive communication structure and guidelines. Centralized communication platform with designated channels for specific topics, projects, or working groups Frequent regular, well-documented (virtual) meetings or calls with clearly defined agenda, responsibilities, and actionable items for follow-ups Common language between all stakeholders (e.g., preclinical scientists, biostatisticians, project managers) by creating an internal glossary or Wiki	Consortium agreement templates (72, 73) or using respective template of institutions (if available) Good Authorship Practice (74) Contributor Role Taxonomy (CRediT) (75) Microsoft Teams, Slack, Google Workplace
(B) Resource Management			
	Time-constrained schedules, lack of buffer time resulting in delays of study milestones. Slow decision-making across multiple labs. Unexpected events or setbacks (e.g., regulatory hurdles, technical failures, etc.). Differences in personnel training and skill level. High staff turnover. Underestimated, hidden, or unforeseen costs. Lack of flexibility in budget allocation. Lack of financial oversight.	Realistic timelines with planned buffer periods accounting for unforeseen delays (e.g., initialization, equipment failure, recruitment of new staff). Early communication with funding bodies, regulatory authorities, or DECIDE-like structures. Centralized decision-making framework. Financial oversight team, study coordinator, or chief risk officer (CRO) for budgeting, contingency plans, and risk assessment. Standardized staff training programs. Effective onboarding and knowledge transfer systems.	
(C) Regulatory Affairs			
	Severe delays in obtaining animal experimentation permits (varying requirements of local regulatory authorities and animal welfare officers).	Early communication with regulatory authorities, animal welfare officers, and DECIDE-like structures as soon as funding is approved. Planning (and budgeting) for financial, regulatory, quality assurance measures or scientific advice. Standardized templates for animal experimentation permit and ethical approval application.

BOX 1 **Illustrative case study—conducting a preclinical confirmatory study.**

A consortium has successfully acquired funding to conduct a preclinical confirmatory study. The aim of this study is to confirm previous findings regarding the efficacy of compound X as a novel intervention against disease Y. To test this hypothesis, the hypothesis-generating *in vivo* experiment of the exploratory study will be conducted again across three different partner laboratories with several adaptations: The confirmatory study will use aged animals of both sexes to better reflect the clinical population. Model induction will follow a new standardized technique to reduce variance in primary outcome measurement. The intervention will be administered acutely rather than prophylactically to improve translational relevance. In addition to saline as a negative control, a clinical gold-standard treatment will serve as an additional comparator.

**Table:** 

Challenges	Solution Strategies
Differences in expertise and resources Lab B and C have never worked with aged animals of both sexes. Lab A and B have never used the new induction technique. Lab A, B, and C vary in animal housing and handling procedures. Lab C uses different equipment than lab A and B.	Initial (virtual) meetings to centrally draft SOPs to harmonize experimental set-up (e.g., animal housing, handling, experimentation protocols, technical SOPs) with regular follow-ups. SOP deviations are documented (e.g., in pre-registration). Contingency plans and quality assurance measures are defined. Experienced personnel from lab A advises lab B and C regarding mouse model. Experienced personnel from lab C visits lab A and B for an in-person workshop to introduce and train new induction technique. Pilot studies across all labs to establish animal model, induction technique and common baseline measurements.
Randomization and blinding inconsistencies Compound X has a distinct color that differs from control treatments, increasing the risk of experimenter bias across all labs. Lab A and B randomize at the level of the experimental unit (the individual animal), whereas lab C randomize at the cage level.	Experimental procedures and data analysis are performed by different individuals to mitigate bias. The randomization method, level, and scheme are defined a-priori (e.g., in pre-registration) and applied consistently across all labs.
Pending regulatory approval Lab A and B are located in different German states, lab C is situated in Switzerland. Animal permits must be drafted for each participating lab considering different local regulations.	Designated time and resources to draft animal experimentation permits. Built in buffer time to mitigate potential delays. Each lab inquires local regulations and uses the relevant permit template (if available) for drafting the animal experimentation application. Early communication with respective regulatory authorities and animal welfare officers.
Data management and analysis issues Each lab uses different file formats and nomenclatures. Data is saved on each respective institutional server. “Outlier Lab”: Experiments conducted at lab B show unexpected adverse effects.	Consortium establishes Data Management Plan (DMP) adhering to FAIR principles as part of the pre-registration to ensure uniform data entry and integrity. Data is additionally stored on a centralized data management platform or repository. Consortium establishes Statistical Analysis Plan (SAP) as part of the pre-registration with clearly defined inclusion and exclusion criteria on a laboratory level. All labs report results according to appropriate guidelines.
Collaboration Project teams across labs have not clearly defined roles and responsibilities for specific tasks. The principal investigator of lab A insists on single last authorship on all related publications. Lab B has a high staff turnover.	Consortium clearly defines responsibilities and action items per lab in a consortium agreement at the beginning of the study. These agreements undergo regular review in (virtual) meetings. Roles and responsibilities can also be pre-registered (e.g., under embargo). Authorship is handled according to Good Authorship Practice. Lab B has well-established communication channels and documentation platforms for effective on- and off-boarding.

### 2.1 Centralized protocols and decision trees

When multiple laboratories participate in a study, the risk of introducing unwanted inter-lab variability increases. This heterogeneity may stem from differences across labs in experimental protocols, animal housing and handling, equipment, or data entry and analysis (10). To minimize undesired variability in the experimental design, data management, and statistical analyses, it is essential to *centrally* draft and distribute comprehensive, clear, and detailed protocol documents and ideally pre-register them (29, 30). These documents should include Standard Operating Procedures (SOPs) to ensure consistent protocol harmonization, compound preparation, *in vitro* and *in vivo* experimentation, as well as blinding and randomization procedures. Ideally, Quality Management Plans (QMPs) (31), Data Management Plans (DMPs), Statistical Analysis Plans (SAPs), and Missing Data Plans should also be drafted centrally prior to project start, then distributed and discussed across all participating labs (32).

It is, however, important to note that not all aspects of a multi-lab study can or should be harmonized. Whereas standardization in animal experimentation can effectively reduce unwanted variability, it may overlook biological variation, thus limiting the generalizability of findings. To address these shortcomings, the concept of systematic heterogenization has been introduced (10, 33). By systematically incorporating sources of biological variability, for instance using animals of different sex or age, varying housing conditions, or changing testing times or methods, this approach is considered to improve study design and result generalizability (33). In other cases, harmonization of all protocols may not be possible, especially when collaborating with international academic labs or industry partners. This includes different laboratory infrastructure and equipment, institutional or regulatory limitations, or financial and resource constraints. It is thus essential to transparently document and communicate these limitations and differences whilst finding a balance between standardization, systematic heterogenization and feasibility.

Moreover, to address possible emergency events such as failure of protocols, allocation disclosure, or compound discontinuation, centralized decision trees, contingency plans, and guidelines should be established early on. However, as workshop participants acknowledged, not all unexpected events can be planned for and even the best-prepared strategies may fall short. It is therefore essential to adapt, evolve, document, and overcome setbacks as they arise without losing focus.

### 2.2 Training and pilot studies

Standardized training sessions and workshops for staff are crucial to ensure rigorous implementation of centralized procedures across all participating labs. These sessions should cover all aspects of centralized workflows with particular emphasis on animal housing and handling, experimental and operational techniques, quality management, and data management. Initial support and on-site visits of more experienced scientists are highly encouraged to facilitate knowledge transfer and consistent training levels among experimenters, particularly for highly specialized methods. If travel is not feasible, the distribution of learning materials, such as videos, serves as an effective alternative.

Pilot studies are useful for validating protocols, standardizing expected drug dose ranges and establishing new methods or models across participating labs. These small-scale experiments help evaluate feasibility and ensure consistent baseline measurements across sites in alignment with the 3Rs principle (*Reduce, Replace, Refine*) (34) before launching the full-scale confirmatory study. It should be noted that conducting pilot studies for *in vivo* experiments can be challenging due to ethical considerations and regulatory constraints. However, if discussed early with regulatory authorities and justified in the sample size calculation of the animal experimentation permit, such studies are still feasible in many cases.

### 2.3 Regulatory affairs

Obtaining animal experimentation permits for a confirmatory multi-lab study was reported as one of the most significant challenges, whether involving different labs within a single federal state (*Bundesland*), across federal states, or international partner labs. This process was further complicated through the absence of a standardized application template for animal experimentation across German states, varying local regulatory requirements, and differing expectations from local animal welfare officers.

Confirmatory multi-lab studies are new to the German research landscape. Due to Germany’s federal system, each participating lab is required to submit its own animal permit application—an inherently time-consuming process. Explaining that these studies are not large-scale replications but rather adequately powered confirmatory studies proved challenging, especially since large-scale replication studies are viewed critically by authorities for ethical reasons. To streamline the process, early engagement with regulatory bodies and close collaboration with animal welfare officers proved beneficial. Consultations and expert support, such as those provided by *DECIDE* and Responsible PrecliniX (*RPX*), were reported to be particularly helpful. *RPX*, a research and support unit at the BIH QUEST Center, assists preclinical research projects to generate patient-informed, robust evidence and has extensive experience in drafting animal experimentation permits in Germany (35).

### 2.4 Documentation

Comprehensive documentation is crucial for managing the complexities of a multi-lab study. To efficiently keep track of protocols, compounds, techniques, blinding, and randomization across labs, transparent and standardized documentation must be established. This also applies to quality management, data management, statistical analyses, and reporting. Several useful guidelines and tools have already been developed to support these latter areas, a selection of which is outlined below.

For quality management, it is recommended to follow GLP (Good Laboratory Practice) (36), PREMIER (Predictiveness and Robustness through Modular Improvement of Experimental Research) (37), and EQIPD (Enhancing Quality in Preclinical Data) (38) quality systems, in addition to the PREPARE guidelines (Planning Research and Experimental Procedures on Animals) (39). Data management is greatly improved by adhering to FAIR (Findable, Accessible, Interoperable, Reusable) principles (40) using standardized formats, a file nomenclature, and a centralized platform aligned with the DMP. Ideally, such a platform should be web-based and secure, supporting collaborative access across labs with role-based permissions. This allows for selective blinding of information from specific users to mitigate risk of bias. Moreover, the platform must ensure data quality, integrate audit trails and version control for traceability, and serve as a central repository for data, protocol documents, and backups—as seen in platforms like REDCap (41). Reporting should follow the ARRIVE 2.0 guidelines (Animal Research: Reporting of *In Vivo* Experiments) (42) and make use of tools such as the Experimental Design Assistant (EDA) (43).

Documentation is equally important for communication. Meeting minutes should be recorded and stored on an accessible, cloud-based server. Given the frequent turnover of scientists in academia, maintaining consistent and well-structured documentation is key to ensure continuous knowledge transfer within the project.

### 2.5 Communication

Effective communication is one of the most critical aspects of coordinating a confirmatory multi-lab study. Specific rules, relationships, and obligations should be established in a consortium agreement along with a clear governance structure to prevent possible conflicts. Together with pre-defined agreements and guidelines on leadership, intellectual property (IP), ownership, and authorship, such frameworks ensure accountability, enable effective decision-making, and balance representation across partnering labs. These commitments must undergo regular review to ensure compliance and must be refined as needed.

Centralized platforms and regular (virtual) meetings are essential for effective communication and trouble-shooting in a multi-lab and multi-stakeholder study. Defined communication channels should inform relevant project members of project status updates, facilitate decision-making processes, and ensure the implementation of centralized procedures across labs. Establishing a common language among all stakeholders—including preclinical scientists, clinicians, patient representatives, biostatisticians, research technicians, and project managers—is challenging yet indispensable for an effective communication throughout the project. While patient engagement is increasingly common in clinical trials, it remains underutilized in preclinical research (44). Confirmatory studies, with their explicit translational focus, offer opportunities for meaningful collaborations provided researchers communicate with other stakeholders clearly and accessibly (45, 46).

## 3 The role of the study coordinator

Confirmatory multi-lab studies hold great promise for generating more robust evidence to guide decision-making in the preclinical trajectory. Nonetheless, they also present an increased workload and diverse challenges in terms of coordination, collaboration, administration, and communication. In clinical research contexts with frequent multi-center trials, roles such as Clinical Research Coordinators (CRC) and Clinical Research Associates (CRA) are common practice. These professionals coordinate clinical trials overseeing data collection, reporting, documentation, and quality control among other responsibilities. In contrast, preclinical research often lacks a dedicated position for study coordination or research management. Of the 12 confirmatory studies funded in the first BMFTR call, nine projects indicated that the leading Principal Investigator (PI) oversaw planning, management, and coordination tasks, often supported by institutional funding. In two other groups, the PI was supported by a postdoc or a clinical trial competence center for project management—and only one group had a designated study coordinator. In alignment with discussions held during the workshop, this highlights the underestimation of the time and resource intensity of such projects.

**Figure 1:**
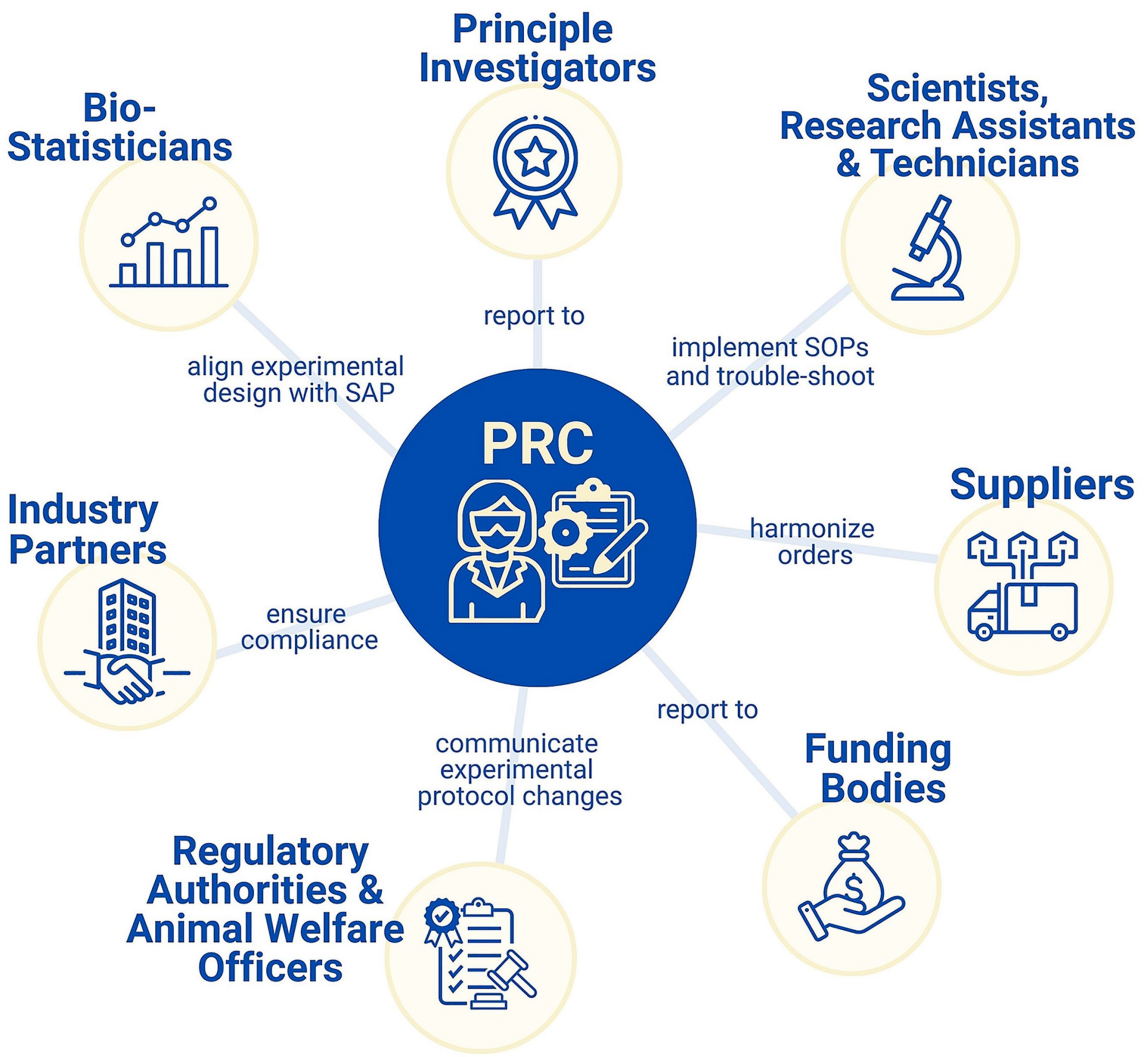
Task and stakeholder mapping of a preclinical research coordinator (PRC). This figure highlights the central coordinating role of the PRC in a preclinical research project, connecting with key stakeholders to ensure effective project execution. Stakeholders are arranged uniformly for visual clarity, however their actual degree of involvement with the PRC can vary considerably.

We thus advocate for new professional figures in the preclinical field, namely preclinical research coordinators (PRCs). Unlike traditional project managers, these roles require strong domain-specific and interdisciplinary knowledge. Specifically, their responsibilities go beyond administrative oversight to include a strong understanding of experimental design, animal models, lab equipment, quality management, GLP compliance, regulatory guidelines, and animal welfare regulations. PRCs serve as a link between all stakeholders of a preclinical study, including scientists, biostatisticians, regulatory authorities, and funding bodies amongst others (Figure 1). As study coordinator, they should be an integral part of the consortium’s coordinating center ensuring effective study oversight. This coordinating center may be one partner lab serving as coordinating lead or may consist of representatives from each partner lab.

To ensure effective project execution as PRC, we propose five actionable recommendations: First, establish a comprehensive study handbook from the start of the project. This living document should be continuously updated and must define all key aspects of the project including experimental procedures, DMPs, QMPs, and contingency plans in case of adverse events. Second, foster clear and consistent communication. As the first point of contact across collaborating labs, the PRC sets up open communication channels and schedules regular meetings with relevant stakeholders. Third, ensure efficient time and resource management throughout the project. The PRC monitors milestones and ensures structure and visibility around deadlines, thus enabling the research team to remain focused and accountable. Fourth, ensure quality control and data management. The PRC maintains centralized platforms for data collection and sharing across laboratories and implements quality control measures throughout the project to identify potential problems early. Lastly, stay flexible and solution-orientated. As not every challenge will have a pre-defined handbook entry, the PRC should not only anticipate unforeseen challenges but remain adaptable and foster open and collaborative problem-solving among stakeholders.

## 4 Discussion

The collective experiences gathered here from preclinical consortia emphasize the importance of confirmatory studies in translational research. Whilst generating more robust evidence, these studies are also highly complex, resource-intensive and bear certain pitfalls. Sustainably integrating confirmatory research into the biomedical landscape will require various stakeholders to adapt and work collaboratively, as outlined below.

### 4.1 Directions for funders and institutions

Funding agencies have yet to fully recognize the value of preclinical confirmatory studies for translational research. Without explicit funder support, adoption of these complex, collaborative study designs will remain limited. We thus advocate for more funding opportunities for both newly emerging preclinical confirmatory infrastructures and existing multi-lab networks and study platforms. A successful example of a sustainable preclinical multi-stakeholder initiative supporting translational research includes ITCC-P4 (47), a non-profit oncology platform that started as a public-private funded project. For funders to effectively assess confirmatory research proposals, we propose a multidisciplinary panel of experts, including domain-specific reviewers, biostatisticians, and experimental design experts to assess the underlying exploratory data and proposed confirmatory study design. Given the complexity of these large-scale projects, funding calls must offer flexibility in timelines, allocation of finances, and allow for international consortia or industry partners. Furthermore, new roles such as preclinical research coordinators (PRCs) should be formally recognized and reflected in funding budgets. Given the current scarcity of these roles, targeted funding is needed to create educational and training resources to build these interdisciplinary competencies. Preclinical confirmatory studies also require stronger institutional support. Translational research centers such as the German Centers for Health Research (DZG) (48) could lead by creating policies and providing infrastructure and resources for such collaborative research. Without coordinated support from both funders and institutions, conducting these studies remains practically infeasible.

### 4.2 Regulatory requirements

To promote the concept of confirmatory multi-lab studies in the preclinical field, clear guidelines and consistent regulations must be established for effective national and international collaborations. This includes, for example, standardized templates for animal experimentation permits and biometric form sheets to reduce administrative workload. To optimize the regulatory approval of a preclinical multi-lab study, we argue for adopting a model similar to that used in clinical trials. In clinical multi-center trials, a leading ethics committee is responsible for the application processing. Other involved committees receive a document copy and must still complete their own ethical and legal reviews, which remains time-intensive. However, the leading committee significantly streamlines the overall process. For preclinical multi-lab studies, each laboratory currently applies for separate animal experimentation permits with varying local requirements, and regulatory authorities rarely communicate with one another. A lead regulatory authority coordinating the animal experimentation process for the entire multi-lab study would significantly reduce delays and bureaucratic burden for all parties.

### 4.3 Gaining recognition in the scientific community

Unanimously, workshop participants emphasized the importance for researchers, both experienced and early career scientists, to recognize the value of preclinical confirmatory research. Improved science communication and visibility—through publications, conferences, workshops, and dedicated training programs integrated into graduate student curricula—could serve as impactful strategies to inform and educate.

There is also a strong need for greater recognition from journals, as many editors and reviewers are unfamiliar with preclinical confirmatory multi-lab study designs. Some may even be dismissive, citing a perceived lack of novelty in confirmatory research or expressing reluctance to publish null results. This lack of recognition ultimately discourages both senior and early-career researchers from engaging in such studies. One way to appeal to more preclinical researchers is for funding agencies to ensure publication in collaboration with journals and publishers and to offer incentives for pre-registration of confirmatory studies. For instance, Registered Reports (RR; a publishing format in which study plans undergo peer-review and approval *before* experiments are conducted) offer an effective way to ensure publication while simultaneously reducing publication bias and reporting bias (49). As an example, the Norwegian funding agency Stiftelsen Dam requires *all* studies to be publicly pre-registered on its registry on the Open Science Framework (OSF) and provides registration templates (50, 51). Moreover, the agency has implemented Registered Reports into their latest research funding program to strengthen transparency, rigor, and reproducibility (52). Another strategy to encourage researchers to publish RRs is through Registered Reports Funding Partnerships (RRFPs). RRFPs are a novel concept where funding agencies and academic journals collaborate to streamline both the grant application and the initial stage of an RR, ensuring eventual publication as an incentive for the scientific community (53).

In addition to pre-registration and RRs, we advocate for a nuanced adoption of Open Science practices to enhance preclinical research quality, transparency, and reproducibility. Through measures like openly accessible standardized protocols and repositories for *all* types of data, scientists can benefit from prior research, save resources, optimize the development of new confirmatory infrastructures, and ultimately accelerate progress in preclinical research.

Confirmatory multi-lab studies offer the potential to generate more robust evidence in preclinical research. Through this, they will putatively promote collaborative efforts, drive medical progress, and reduce research waste, ultimately enhancing translation from bench-to-bedside. However, herein described studies are highly complex, time-consuming, and resource-intensive, and require more rigorous planning, collaboration, and oversight than common exploratory single-lab studies. We thus propose actionable strategies and advocate for new professional roles such as preclinical research coordinators (PRCs) and for the involvement of diverse stakeholders to enhance preclinical evidence synthesis. While we acknowledge that our recommendations bear certain limitations and are not fit-for-all-purpose, we stress the importance and necessity of confirmatory preclinical research to drive innovative biomedical advancements.

## Data Availability

The datasets presented in this article are not readily available because the datasets generated and/or analyzed during the current study, including presentation slides and collaborative workshop notes, contain identifiable project-level information from multiple research consortia and are not publicly available due to confidentiality agreements. De-identified summaries of the themes and findings derived from the workshop discussions are available from the corresponding author upon request. Requests to access the datasets should be directed to sophia.rotter@charite.de.
